# Striving for Confidence and Satisfaction in Everyday Life with Chronic Obstructive Pulmonary Disease: Rationale and Content of the Tele-Rehabilitation Programme >C☺PD-Life>>

**DOI:** 10.3390/ijerph16183320

**Published:** 2019-09-09

**Authors:** Charlotte Simonÿ, Claus Riber, Uffe Bodtger, Regner Birkelund

**Affiliations:** 1 Department of Physiotherapy and Occupational Therapy, Slagelse Hospital, Faelledvej 7, 4200 Slagelse, Denmark; 2 Institute of the Regional Health Services Research, University of Southern Denmark, 5230 Odense, Denmark (U.B.) (R.B.); 3 Department of Respiratory Medicine, Naestved Hospital, Ringstedgade 61, 4700 Naestved, Denmark; 4 Department of Respiratory Medicine, Zealand University Hospital, Sygehusvej 10, 4000 Roskilde, Denmark; 5 Department of Health Research, Lillebaelt Hospital, University Hospital of Southern Denmark, Beriderbakken 4, 7100 Vejle, Denmark

**Keywords:** e-health services, chronic pulmonary obstructive disease, intervention, telerehabilitation

## Abstract

*Background:* More feasible rehabilitation programmes for patients with chronic obstructive pulmonary disease (COPD) are warranted. Even so, still in its infancy, telerehabilitation to COPD patients reveals promising results, wherefore it is anticipated to contribute significant value to the current challenges of rehabilitation to these patients. To expand useful knowledge in the field, more sophisticated telerehabilitation interventions must be developed and appraised, but first and foremost, thoroughly described. *Aims and methods:* The aim of this article is to give a detailed description of the rationale and content of the **>C☺PD-Life>>** programme, within the bounds of the checklist of Template for Intervention Description and Replication (TIDieR). *Approach:* **>C☺PD-Life>>** is a telerehabilitation programme for COPD patients delivered as a study intervention by an interprofessional team of clinicians collaborating from both the hospital and the municipal healthcare system. Making use of two-way audio and visual communication software, 15 patients participated in the intervention via a tablet computer from their private setting. The programme was a six-month-long empowerment-based rehabilitation that aimed to support COPD patients in leading a satisfactory and confident life with appropriate physical activity and high disease management. *Conclusions:* A long-term interprofessional cross-sectoral telerehabilitation programme has been justified and described. The intervention was tested in 2017–2018 and the qualitative appraisal, along with an analysis of case-based measurements of development in physical capacity, COPD Assesment Test, and health management, is currently under production.

## 1. Introduction

To optimize future rehabilitation to patients with Chronic Obstructive Pulmonary Disease (COPD), it is imperative to target the WHO definition of supporting disabled people: *“*to attain and maintain their maximum independence, full physical, mental, social and vocational ability, and full inclusion and participation in all aspects of life” [1] (p. 3). New programme models that improve uptake, access, as well as coordination, are required to accommodate the complex and individual requirements of COPD patients. 

Typically, rehabilitation involves eight to twelve weeks of twice-weekly supervised exercise training in a group setting, as well as education regarding COPD self-management. However, despite the compelling evidence [2,3] attendance and completion in standard pulmonary rehabilitation remain low. Barriers to this include, for example, lack of belief in potential benefits, conflict with daily routines, limiting comorbidities and transport issues [4,5,6,7]. 

Moreover, complicated pathways for the COPD patients across healthcare sectors as well as complex communication with interdisciplinary professionals are shown to confer unmet needs for support in every day life, unnecessarily prolonged care and treatment, and poor prevention of stressful and expensive hospital admissions [8,9,10].

Recent international minor scale studies reflect that tele-based rehabilitation solutions to COPD patients are currently expanding with promising results. Programmes consisting of physiotherapist-guided exercise are shown to be both feasible, safe [11] and capable of supporting improvement in the physical condition, quality of life and self-efficacy, and moreover, to elude aggravation in disease status [12,13,14]. 

As argued by The United Nations Convention on the Rights of Persons with Disabilities (UNCRPD), assistive technologies are recognized as a human right that allows equal opportunities to be supported to maintain or improve functioning and independence and thereby promote well-being [15]. Therefore, contribution to further development and refinement of the promising scope of the already commenced telerehabilitation solutions should be assigned to the multi-challenged COPD patients. In this spirit, the telerehabilitation intervention **>C☺PD-Life>>** was developed to offer to COPD patients a broad coherent and well-coordinated rehabilitation delivered by interprofessional clinicians from both the hospital and the municipal healthcare system. The aim was to provide support to the patients in leading a satisfactory life with appropriate health and disease management and confidence, despite the disease. 

Since detailed descriptions of interventions are imperative for reliable implementation into clinical practice [16,17], this paper describes the **>C☺PD-Life>>** intervention guided by the 12-item checklist from the Template for Intervention Description and Replication (TIDeR). The specific elements are delineated to promote transparency on reporting of the intervention [16].

## 2. >C☺PD-Life>> (COPD: Chronic Obstructive Pulmonary Disease)

The intervention was entitled **>C☺PD-Life>>** to reflect that the concept aims to support people with COPD in leading a satisfying and confident daily life with high health and disease management. In the logo, the arrows and the increased blue color symbolize an expected improvement in daily living and rise of oxygen in the body and life of the participants.

### 2.1. The Rationale of **>C☺PD-Life>>**

**>C☺PD-Life>>** was developed as an intervention for a pilot study with inspiration from the framework of complex interventions [18,19]. 

It was desired to deliver a well-coordinated, cross-sectoral, long-lasting, easily accessible, flexible and individually supportive rehabilitation programme for COPD patients. This was expected to subsidize them in improving health and disease management.

#### 2.1.1. An Empowerment-Based Intervention 

Regardless of the level of severity of clinical presentation, an empowerment approach is shown to be useful in supporting patients in improving the ability to identify, express, and share unique and unexpected difficulties related to living with a disease [20,21,22]. Therefore, we based the intervention on this philosophy.

In his work “Pedagogy of the oppressed”, the Brazilian educator and philosopher Paolo Freire emphasized that a learner, by trustful and careful dialogue, should be supported to gain the capacity to adapt to reality and to make choices that transform this reality. To Freire, the dialogue is not only required between educator and learner but also within a group of learners. Through dialogue, people become able to describe and interpret their world. He considered dialogue as an existential necessity which could only truly exist on the basis of love, hope, humbleness, equality, and ability to be critical [23]. He argued that dialogue-based education provides a movement from naïve consciousness to critical consciousness, leading to critical action [24]. This critical consciousness is characterized by depth in the interpretation of personal problems, and Freire stated that: “The important thing is to help men help themselves, to place them in consciously critical confrontation with their problems, to make them the agents of their own recuperation.” [24] (p. 12). Hereby, the critical consciousness requires people to take responsibility, to practice dialogue and be truly open towards the possibilities and revisions that their problems accentuate. This frees people and enables them to act in accordance with what is important in their own lives [23,24]. It was our intention to base the rehabilitation in **>C☺PD-Life>>** on this empowerment philosophy in order to encourage the critical consciousness of the participants. Accordingly, the respectful dialogue was the chosen communication, both in the group sessions and in the individual consultations.

#### 2.1.2. A Rehabilitation Intervention for Patients with COPD 

Within one month before starting each of the two courses of **>C☺PD-Life>>**, patients with COPD who were referred to standard out-patient pulmonary rehabilitation in Slagelse Hospital, Denmark, were invited to participate. Inclusion criteria were: a doctor’s diagnosis of COPD, referred by a general practitioner or hospital physician for pulmonary rehabilitation, a citizen of Slagelse Municipality, and fluent in Danish. Exclusion criteria were: critical other illness such as, but not limited to, severe heart failure, endstage renal or hepatic failure, metastatic cancer, age under 18 years, inability to give informed consent. 

Engaged patients were orally introduced to the intervention in a quiet room at the hospital, and if desired, close relatives were also a part of this introduction. They were given written information, and days at home, to consider participation. If further information was needed, this was provided by cell phone on the patients’ request. They were specifically informed that participation was voluntary, that they could withdraw from the study at all times without adverse complications for their care, and that if they preferred standard pulmonary rehabilitation, it was fully accepted. 

In total, 28 eligible patients were informed, and 16 gave written informed consent. However, one withdrew the consent within 5 weeks because of lack of time due to unexpected workload, thus 15 patients (7 women and 8 men) accepted participation and completed the programme (see Table 1). The 12 declining patients refused participation due to challenges with diseased spouses, lack of energy or uncertainty of the benefit of the programme: fear of not being able to manage the technology, not having the discipline to attend the sessions from home, and fear of exclusion from getting a social relationship with peers.

The intervention study was approved by the Ethical Committee (SJ-559) and the Danish Data Supervisory Committee by Region Zealand (J. Nr. REG-071-2016), and their guidelines along with ethical principles of the Declaration of Helsinki were followed [25].

Each participant was offered a tablet computer (iPad Pro 9.7 32GB 4G) for the entire intervention period. A video software programme, KMD Viva, allowing two-way audio and visual communication, was used. The microphone, speakers, and camera on the tablet made the communication possible. The online connection was established via SIM-cards. The participants had no expenses using the equipment. 

For exercise sessions, the participants were handed: a step bench, rubber bands (3 different resistance) and two dumbbells (0.5–10 kg each). Other exercise equipment used included a dining table chair (not delivered). 

### 2.2. The Content of **>C☺PD-Life>>**

We designed the programme to follow the national guidelines for pulmonary rehabilitation in Denmark [26] and with regard to existing evidence in the field. Here we especially focused on what was essential to COPD patients in rehabilitation. In addition, previous findings from a preliminary study of COPD patients’ experiences of their participation in standard pulmonary rehabilitation [27,28] were considered along with practical issues.

By use of two-way audio and visual communication software, **>C☺PD-Life>>** was delivered as online rehabilitation into the patients’ own setting. Everyone attending could see and talk to each other. Three to five group sessions were held every week for six months. Moreover, individual consultations were provided according to each participants’ individual profile. The content is further described in the section “The course of **>C☺PD-Life>>**”. 

To appraise the intervention, it was investigated, within a qualitative study frame, what it meant to the participants to receive **>C☺PD-Life>>**. This was performed by combined participant observations and individual interviews [29]. The participation was registered along with the kilometers of saved transportation and the participants’ profile for COPD-related hospital admissions. In addition, the development of self-management [30], physical capacity [31], and status in lung function [32] were examined. 

#### 2.2.1. A Multifaceted Rehabilitation Programme Provided by A Healthcare Team

An eight-member inter-professional healthcare group was chosen to design and provide **>C☺PD-Life>>**. The members were specifically allocated to fulfill the programme besides their daily clinical work. All were skilled clinicians within the rehabilitation of COPD patients. Representing both the primary and the secondary healthcare sector, the team included: two nurses, two physiotherapists, two occupational therapists, a daily living consultant and a specialized nurse consultant from the regional Specialized Center of Lung Diseases. The team initially planned the programme in detail and made a two-week feasibility test with three patients before starting. 

Subsequently, the team offered the programme within a continuously coordinated collaboration applying the empowerment approach. 

As shown in Box 1, the programme included varied group-based and individual offers delivered from the interprofessional team.

Box 1 **The content of the >C☺PD-Life>> programme.**

**Physical exercise training**

Three times a week—Monday, Wednesday and Friday, group-based supervised exercise with a physiotherapist was offered from 10 am to 11.15 am. A 26-week group-based exercise plan was planned in detail for every session. If required, individually supervised training sessions were held.

The exercise training consisted of endurance and resistance training [3,33]. The sessions included group-based guidance that invited all to participate in exercises demonstrated and corrected by the physiotherapist. When an individual had special demands, they were guided to perform the exercise with regards to their personal strengths, limits, and goals.

Initially, instructions were given of breathing technics, and continuous support in performing this adequately was given during exercise. The sessions were scheduled to contain 10 min of warm-up, 50 min of endurance or resistance training, followed by 5 min of warm down. All sessions of the exercise were finished with 5–10 min of stretching and relaxation combined with deep breathing training.

The resistance training was progressive and dynamic with 3 sets of 10 repetitions maximum. Exercises focused on both upper and lower limb and core as biceps, triceps, quadriceps etc., and included the use of dumbbells, rubber bands a step bench or a chair. The endurance training was interval-based and progressive with intensity from 60%–80% of maximum working capacity with regards to each individual [33,34]. Exercises like walking, arm rowing, etc., were performed. There was a continuous shift between emphasizing either endurance or resistance training during each exercise session.

The above-mentioned exercise principles were used exclusively for those participants who could contribute to this. A careful and thorough assessment of contraindications was taken, especially if participants simultaneously had other significant co-morbidities, such as heart disease, osteoporosis, metabolic diseases, etc. Both in relation to the endurance and the resistance training part, the physiotherapists would adjust the workout load continuously in order to ensure that the individual participant’s working capacity fitted the participant’s development and health status [33]. If participants became hyperinflated, they were guided to work on focused breathing techniques and if necessary relaxation during exercise.

 

**Physiotherapy**

Besides the supervised exercise, the participants were also offered sessions of education and individual consultations with the physiotherapists. This mainly concerned consultation according to their goals including adjustment in techniques and workload during exercising, injury treatment, instructions and guidance in performing daily activities in a more energy-conserving matter. Predefined educational topics were: breathing techniques, e.g., instruction in the use of the positive expiratory pressure (PEP) device, physical activity and training principles, lung anatomy and COPD, incontinence and pelvic exercise, maintaining physical activity and pain management [2,28]. During these educational sessions, the techniques were trained with intensive guidance from the physiotherapist.

 

**Nursing**

As argued in the research of nursing to COPD patients, the two nurses collaborated in performing a holistic and patient-centered-care on the basis of the patients’ individual needs in daily living with COPD [35,36,37]. Moreover, this was in line with the overall empowerment approach in the programme. Based on preliminary interviews with the participants, they continuously supported them via on-going consultations. Either the consultations were scheduled or ad-hoc required by the participants or by referral from the colleagues from the rehabilitation team.

The nurses held educational sessions approximately once a month. Their topics were: patients’ needs, expectations and self-care, the physiology of the lungs, management of medicine and inhalation techniques, management of dyspnea and panic, cohabitation and sexuality, existential and palliative matters, smoking cessation, social options and issues related to additional illness (apnea, incontinence, diabetes, osteoporosis, malnutrition etc.) [9,27,28,38,39,40,41,42,43]. One of the nurses was available for individual consultations most days during the programme.

The participants were also given two sessions of education by a specialist nurse from Region Zealand’s Specialized Center of Lung Diseases. Here, they were introduced to volunteer networks and to the regional center of pulmonary disease management in its own (organisation and history) and how it could be relevant to use. Furthermore, the participants were introduced to the rights and the legislation as citizens with lung disease, e.g., according to travel and transport issues, etc.

In addition, they were invited to consult the specialist individually if desired.

 

**Guidiance in daily living**

The daily living consultant offered group-based education sessions and individual support according to important knowledge about healthy nutrition, e.g., the importance of taking a diet with lots of proteins and fibres, exercising etc. [44]. The participants were guided about what they must do if they had had a major inappropriate weight loss or were overweight.

 

**Occupational therapy**

The occupational therapist performed patient-tailored guidance by the use of the Canadian occupational performance measure (COPM) [45,46].

Furthermore, four educational sessions were held with the focus on energy conservation principles in relation to indoor and outdoor activities [47] and aspects of dysphagia [48].

Efforts were made to guide the participants individually with concern to what is of specific importance to them in their daily living.

 

**>C☺PD-Life>> cafés**

Every second week, an informal session called **>C☺PD-Life>> cafés** was arranged. The café made it possible for the participants to talk with a member of the provider team and the peer participants about what they would like relevant to the disease, while sitting in a comfortable chair, having a cup of coffee, tea, etc.

#### 2.2.2. The Course of **>C☺PD-Life>>**

**Figure 1:**
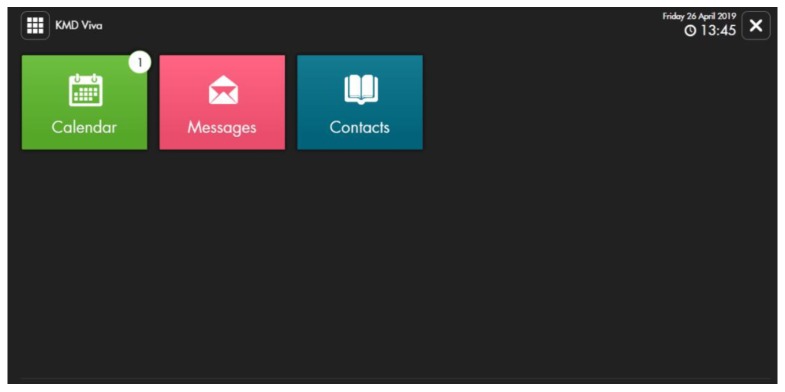
An example of a front screen with different applications; e.g., calendar, messages, and contacts.

**Figure 2:**
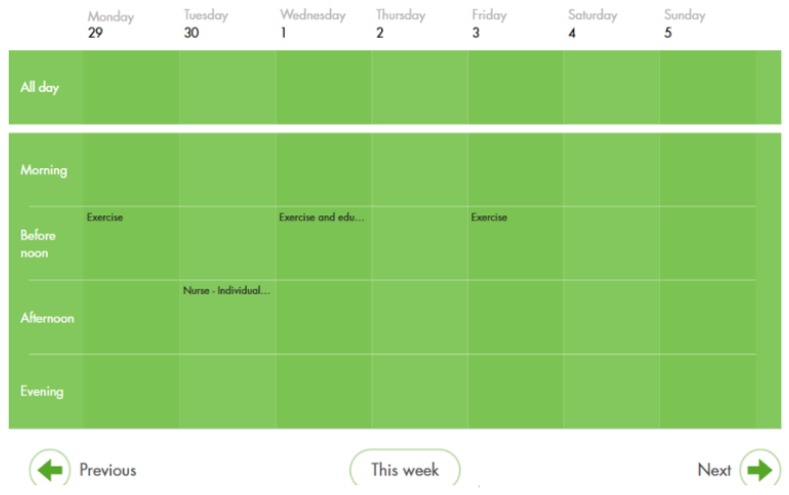
An example of how the calendar showed all planned actions on a daily, weekly or monthly basis.

The participants attended sessions from their home via a tablet computer or desktop. Access was gained by use of personal security login codes. The content of the programme was scheduled in a calendar in the software visible for the participants (see Figure 1 and Figure 2). 

The provider team worked as a unified entity in a well-coordinated collaboration while closely following the participants. The participants were supported to achieve a critical consciousness in decisions, thus being subsidized in exploring and continuously bettering their understanding of the content of their requirements. The providers motivated the individual participants to maintain new sustainable initiatives and improvements in their daily life. 

**Figure 3:**
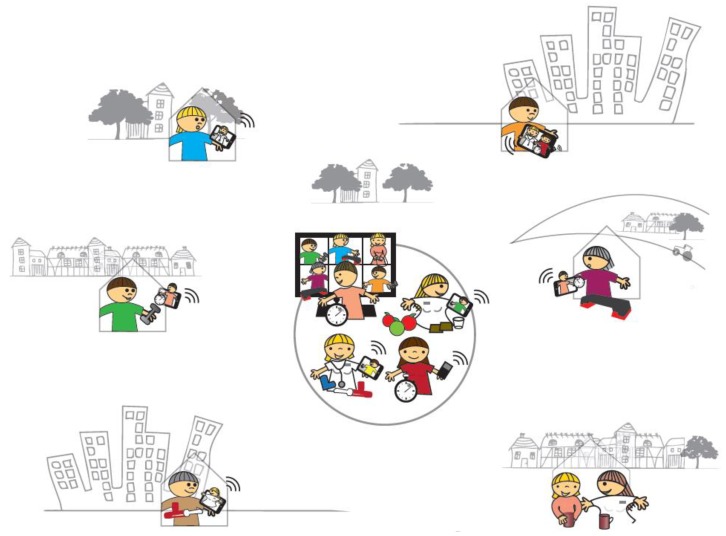
**>C☺PD-Life>>**—Pulmonary rehabilitation brought into COPD patients’ setting. In **>C☺PD-Life>>**, people with COPD participated in pulmonary rehabilitation directly from their own home via a tablet computer for 26 weeks. The software programme allowed the patients to participate in online interactive video contacts with the providing team and their peer patients. A co-operating interprofessional provider team representing both the hospital and the healthcare section in the municipality, consisting of nurses, physiotherapists, occupational therapists, a daily life consultant and a specialized nurse consultant from the regional Specialized Center of Lung Diseases offered the programme. The providing team had big screens so that they could clearly see all participants.

Mondays, Wednesdays, and Fridays, 45–75 min long group-based physiotherapist-guided exercise sessions were held. In addition, one session of group-based education (selected topics) was offered every week along with café meetings every second week. The educational sessions lasted for 45–60 min and followed an exercise session superseded by a 10–15 min break. On those days, the training sessions were shortened to avoid long sessions. The participants received information and teaching about a predefined topic by either one or more from the provider team. The sessions were dialogue-based and the participants’ querying for other relevant topics were met. The participants were able to invite their relatives if wanted. The participants were offered to have a shared session with particular involvement on their relatives’ part, managed by the two nurses and one physiotherapist. Furthermore, individual consultations with represents from the rehabilitation team were offered (see Figure 3). Consecutively intense individual guidance was given within group-based or individual consultations to accommodate the specific goals, development, and needs of each participant. Individual consultations were planned in collaboration between each participant and the provider team for immediate or later settlement, according to the particular situation. During daily work time, ad hoc query for consultations was accessible. This was carried out by telephone calls to the team. If relevant, representatives from the provider team visited the participants in their homes. 

#### 2.2.3. An Introduction Day

Initially, participants and the provider team met for four hours at the hospital. Relatives were also welcome to attend. 

All participants underwent an initial spirometry and assessment of exercise capacity by a 6 min walking test (6MWT) [31] or 30-s sit-to-stand test (30-STST) [49], in accordance with usual practice in pulmonary rehabilitation. In 6MWT, heart rate and oxygen saturation were monitored, and the participants rated their perception of dyspnea on the Borg CR-10 dyspnea scale before and after 6MWT. Midway and finalization 6MWT or STS tests were completed at week 13 and 26 of the programme at the hospital followed by individual evaluation with each participant.

Instructions on the use of the technical equipment were provided (ongoing technical support was given either by phone or physical meeting in the participants’ homes as demanded).

Individual consultations concerning e.g., expectations, goal setting, and physical tests were performed. Based on the introductory test and consultations, the provider team created action plans to support and supervise each individual participant by the empowerment approach.

### 2.3. The Setting

COPD patients from the local area of the hospital and municipality participated from their private home or work. The video meetings started at scheduled times, which were agreed in advance and booked into the system by the professionals. The participants entered the sessions by a personal log-on code. The attendees were all able to interact with visual and auditive contact. The provider team used big screens for group-based sessions, which allowed good visual contact. 

### 2.4. A Twenty-Six-Week-Long Programme

Two identical programmes of 26 weeks were completed. The first programme had nine participants, running from April 2017 to October 2017. The second programme had six participants, running from October 2017 to April 2018.

The average expenditure of time was approximately 15 h of clinical work per week provided by the team. 

### 2.5. Tailoring the Participating Patients

**Figure 4:**
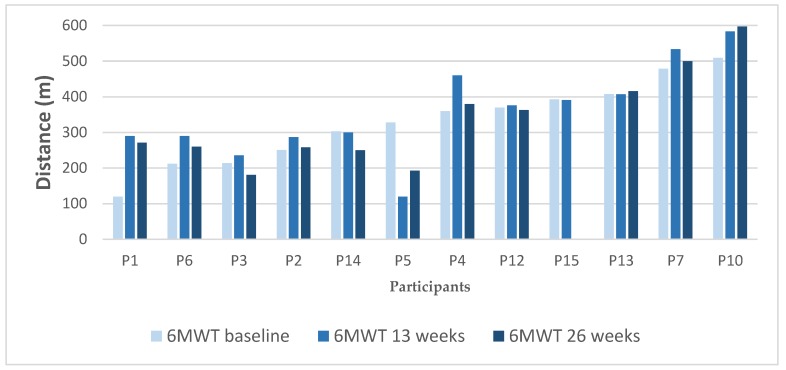
6 min walking test (6MWT) results during the intervention, ordered after baseline results (from lowest to highest).

In collaboration with the providing team, each participant attended the sessions that were deemed appropriate according to his or her individual goals and capability. Results from 6MTW (see Figure 4) or STS were was considered at an individual level in the following exercise guidance.

The development in the change in 6MWT is variable. Generally, the spread is large, i.e., scores ranging from 120 m to 509 m at baseline, 120 m to 597 m at 13 weeks follow-up and 193 m to 597 m at 26 weeks follow-up. In accordance with reference to a minimum clinical important difference being ≥30 m [31], the changes in 6MWT during the intervention can be divided into three groups: three (27%) had clinical important improvement, five (46%) had no change and three (27%) had clinical important deterioration. This indicates that only three worsened while the overall change in physical capacity includes either keeping status or even improvement. 

**Figure 5:**
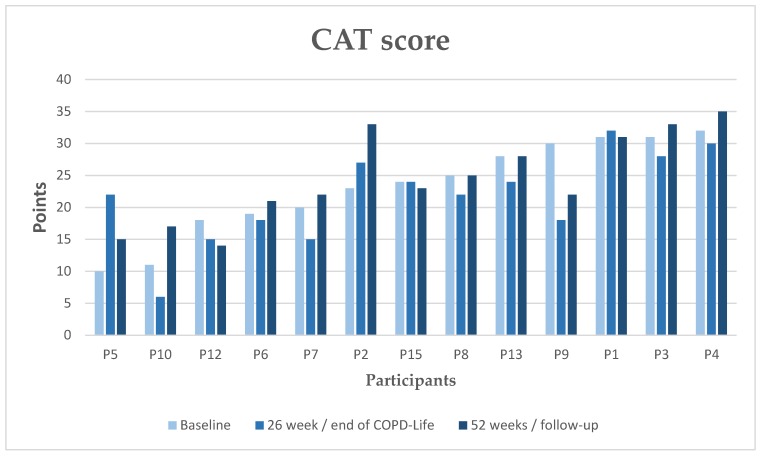
Participant’s results in COPD Assessment Test (CAT) score before and after **>C☺PD-Life>>** and at a 52-week follow-up, ordered after baseline results (from lowest to highest). Two patients, P11 and P14, did not fulfil the questionnaire correctly so their data is excluded.

To monitor change in participants’ symptoms burden, a COPD Assessment Test (CAT) [32] was performed at intervention start (baseline) and at end of the intervention. Furthermore, a 52-week follow-up from baseline was performed. The participant’s CAT score and development of this are shown in Figure 5.

As shown in figure 5, the participants reported a large variation in their CAT scores both at baseline (range from 10 to 32 points), at the end of the intervention (range from 6 to 32 points) and at the 52-week follow-up (range from 14 to 35 points). 

Changes in CAT were identified in three groups in line with recommendations for the minimum clinical important difference being ≥2 units [32]. From the start to finishing **>C☺PD-Life>>**, eight (62%) had clinical important improvement, three (23%) held status and two (15%) had clinically important aggravation. This reflects that the intervention is likely to be considered as effective to the majority of the participants. From baseline to the 52-week follow-up: two (15%) participants had clinically important improvement, four (31%) participants had no clinically important change and seven (54%) participants had a clinically important aggravation. In this light, the long-term effect of **>C☺PD-Life>>** seems low [32]. However, larger populations are needed to examine the impact on CAT of this telerehabilitation program. 

### 2.6. Modifications during the Intervention 

The overall programme for the participants was not modified during pilot trials. However, formal scheduling of team meetings among the providers was added during the first programme. 

### 2.7. Plans for Ensuring Fidelity and Monitoring Adherence in **>C☺PD-Life>>**

The providing team was initially trained in the management of the KMD software. They collaborated in the planning and performance of **>C☺PD-Life>>**. To ensure fidelity, the leader of the intervention continuously supervised the team. One month after the initial start-up a status meeting was held. Weekly written status reports were sent to the team, and status meetings were conducted every third month. Each team member had evaluation meetings with the leader and their daily manager during the intervention period. 

To follow up on the receipt of the participants, their attendance was registered from the KMD software and by the providing team in a shared protocol.

### 2.8. The Extent of **>C☺PD-Life>>**.

All performed sessions were registered. Besides few reschedules due to sickness among the providing team, 14 group sessions out of 224 were canceled because no participants attended. Infrequent individual consultations were held over the telephone according to the participants’ preferences.

Throughout both courses of **>C☺PD-Life>>**, approximately 1005 h of communication between participants and the provider team had been conducted. 658 contacts were completed via KMD Viva. 

**Figure 6:**
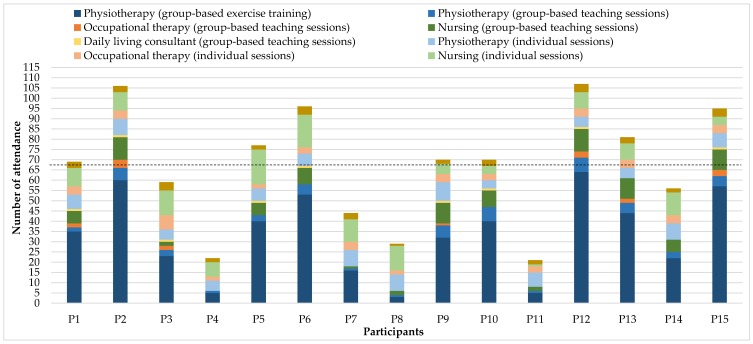
Participation in **>C☺PD-Life>>**.

As shown in Figure 6, the participation during intervention was relatively high and with great variations. On average, each participant attended the programme by 67 contacts, equivalent to approximately 71 h of interaction. The majority of the participants had the most contacts through the group-based exercise training with physiotherapists, followed by individual and group-based contact with the nurses. Moreover, most participants had a relatively large share of individual conversations or training with the physiotherapists. They had slightly less contact with the occupational therapist and the daily living consultant both in group-based education and in the individual conversations, see Figure 6. During the programme, there was a continuous dialogue between each participant and the providing team evaluating their attendance with regards to their current status and goal-setting. Accordingly, adjustments for their individual focus, development and programme participation were made. 

On average, the participants saved transport of 1516 km during the **>C☺PD-Life>>** course, i.e., for both exercise training, teaching and individual conversations. There is great variation in saved transport for the individual participants, reporting from 105 km to 4256 km.

The participants attended the intervention with great variations. Their preferences reflect their individual situation and queries. The major part of attendance was to the group-based supervised exercise training, with an average of 33 times, equivalent to participation in training 1.5 times a week. The average attendance in the group-based educational sessions was 12 times and the average participation in the individual offers was 22 times.

## 3. Discussion, Limitations and Suggested Modifications–Lessons Learned

Guided by the TIDieR checklist, this manuscript provides a detailed description of the telerehabilitation intervention **>C☺PD-Life>>**. First, the intervention is expected to enhance the support of COPD patients in leading daily life with improved confidence, satisfaction, and well-being, in line with international policy [1,15]. Second, the investigation of the appraisal of the intervention is expected to shed new light and add important knowledge into the research field, concerning approaches and planning for future pulmonary rehabilitation programmes, allowing implementation and replication by researchers and clinicians for coming studies.

As argued by Desmond et al. [50] and Ludvigsen et al. [51], the patient perspective is involved in both design and content of the intervention, which gives a unique opportunity to target the programme according to what is essential for people with COPD, and thus to better meet the recommendations for rehabilitation from WHO. This contributes to central knowledge of future planning of the rehabilitation efforts.

Involvement in the early phase of the study of the provider team, both according to the content and design of the program, led to the intervention being on track from the very start. This is in accordance with the recommendations for complex interventions [19]. Furthermore, it meets the significant assistive technology-based intervention strength described by Yang and Penda [52]. They conclude that a robust process of the design is the key to improve the outcomes of the small and medium-sized enterprises of tele health-care solutions [52]. 

Time, especially in the early phase of the programme, was required to ensure managing the technological equipment for both staff and patients [15]. Nevertheless, the average weekly 15 h of clinical work provided during the intervention must be considered to be cost-effective. Moreover, the saved kilometers should be taken into account.

The inherent flexibility of the tele-based solution enables patients to easily collaborate with professionals regardless of distance, other tasks in everyday life and challenges due to dyspnea, as well as other physical inabilities. In addition, the well-known fluctuations in the symptoms of COPD [8,40] can more easily be accommodated by such flexible and interactive communication. Such flexibility is anticipated to improve feasible healthcare support to COPD patients in many settings worldwide. 

The relatively high attendance during the intervention along with the inherent flexibility are fortunate factors. However, considerations must be given to the notable variations in the individual use of the specific parts of the offer. Further analysis is required to understand this better.

Offering a shared session with the involvement of close relatives were only performed during the second course of the intervention. The patients in the first course did not find it relevant. However, relatives seem to be in need of involvement [53] and this should be addressed better in future telerehabilitation.

Goal setting and evaluation is expected to require continuous follow-up. This should be performed with respect to the individual patient’s autonomy and status [2,3]. From the first intervention course, we learned that to achieve the best possible collaboration within the providing team, structured meetings are imperative. Thus, we established a one-hour online meeting every fortnight with the providing team. Here they reviewed the patients’ goals, discussed their individual situations and made action plans in each case.

Providers for future similar interventions are recommended to, at the same time, endeavor an outreached and empowerment-based role in order to accommodate individual needs and requirements from patients. Besides, close interdisciplinary cooperation as well as generally strong communicative skills should be considered particularly important, among providers, since healthcare professionals have a considerable role to facilitate and give feedback on the use of assistive technology [54].

To our knowledge, cross-sectoral interventions within COPD rehabilitation are, as yet, under-examined. The complexity of the differences within the primary and secondary healthcare sectors may cause this [8,10,55]. Nevertheless, developing well-coordinated pathways for chronic patients seems dependent on such collaboration.

The current methods are not specific to the Danish healthcare system or Danish patients, and our model should be readily transferable to other healthcare settings.

## 4. Conclusions

The current need for optimizing beneficial rehabilitation for COPD patients indicates a warrant for more accessible, sustainable, individualized and equitable options. Since studies of telerehabilitation reveal promising results, further development and investigation are relevant. The TIDieR checklist provides a useful framework for describing the rationale and detailed content of the long-term cross-sectoral intervention **>C☺PD-Life>>**. This critical, yet under-utilized step in the intervention development process provides the rationale and content for the intervention, which has been investigated and will be reported in a qualitative appraisal of the participating patients’ experiences and case-based analysis of measurement in development in their physical capacity, CAT-score and disease management. The present intervention has the potential for replication in other clinical settings. However, further refinements by clinicians, leaders, and researchers must be considered.

## Figures and Tables

**Table 1:** Characteristics of the participants in **>C☺PD-Life>>**.

Participant	Gender	Age (Years)	Married/ Cohabitated	Status of Employment	FEV_1_ (% Predicted)	Smoking
**P1**	F	60	−	Work capacity evaluation	22	S
**P2**	M	74	+	Retired	63	FS
**P3**	M	45	+	Retired	25	FS
**P4**	M	50	+	Retired	62	FS
**P5**	F	83	−	Retired	117 *	FS
**P6**	F	55	+	Retired	21 **	FS
**P7**	M	63	+	Work capacity evaluation	26	S
**P8**	F	62	−	Retired	50	S
**P9**	F	64	−	Retired	26 **	FS
**P10**	M	65	+	Employed	47	FS
**P11**	M	58	−	Retired	28 **	FS
**P12**	F	61	−	Retired	34	FS
**P13**	F	67	−	Retired	41	FS
**P14**	M	62	−	Retired	27	FS
**P15**	M	63	+	Retired	51	FS
**MEAN**	53.3 % (M)	62			42.6	

* Severe centrilobular emphysema with impaired diffusion capacity. ** Long-term oxygen treatment; M: male; F: female; mMRC: modified Medical Research Council dyspnea scale; FEV_1_: Forced expiratory volume in 1 s. S: Smoker. FS: Former Smoker.
